# Curcumin and Curcumin-Loaded Nanogel Induce Apoptosis Activity in K562 Chronic Myelogenous Leukemia Cells

**DOI:** 10.22086/gmj.v0i0.921

**Published:** 2018-02-23

**Authors:** Sepideh Khatamsaz, Mehrdad Hashemi

**Affiliations:** ^1^Department of Molecular and Cellular Sciences, Faculty of Advanced Sciences & Technology, Pharmaceutical Sciences Branch, Islamic Azad University, Tehran -Iran (IAUPS); ^2^Department of Genetics, Tehran medical sciences branch, Islamic Azad University, Tehran, Iran

**Keywords:** Curcumin, Chronic Myelogenous Leukemia, Nanogel, Chitosan, Stearate

## Abstract

**Background:**

Chronic myeloid leukemia (CML), a hematological cancer of stem cells, is caused by the activation of oncogenic factors alone or/with inactivation of tumor suppressor genes. Curcumin is a hydrophobic polyphenol and the main compound of turmeric, which has been used in daily diets for many years. It is also a safe drug. Nanogels and nanobiotechnology have important roles in the diagnosis and treatment of diseases and drug delivery.

**Materials and Methods:**

To prepare the nanodrug, chitosan nanogels were prepared in 1% acetic acid and cross-linked with stearate by 1- ethyl- 3 (3-dimethylaminopropyl) carbodiimide (EDC) and N-hydroxysuccinimide (NHS). Subsequently, curcumin was loaded in the chitosan-stearate nanogel. Physical and morphological characteristics of the nanodrug were determined by transmission electron microscopy (TEM), dynamic light scattering (DLS), and Fourier transform infrared spectroscopy. Different nanodrug concentrations were prepared and evaluated on the K562 CML cell line. The apoptotic activities of curcumin and nanodrug on the cells were detected by flow cytometry, MTT assay, and trypan blue staining.

**Results:**

DLS revealed that the size of the nanodrug was 150 nm, which was confirmed by TEM. The half maximal inhibitory concentration (IC50) values of curcumin and nanodrug were 50 and 25 μg/ ml, respectively P < 0.05). Apoptosis of the K562 cell line occurred at 48 h post-treatment with 25 μg/ml curcumin and 12.5 μg/ml nanodrug.

**Conclusion:**

The increase in the cytotoxicity of curcumin and nanodrug was directly related to the drug concentration and time. The nanodrug exhibited more cytotoxic effects on the vital capacity of the cells and stimulated more apoptosis compared with curcumin alone.

## Introduction

Chronic myeloid leukemia (CML) causes a cytogenetic abnormality known as Philadelphia chromosome and results in the uncontrolled growth of bone marrow cells [1]. To optimize conventional cancer treatment, researchers are searching for appropriate complementary medicine to improve treatment performance. Unlike the first-line drugs for cancer treatment, herbal compounds can affect cancer in several ways via valuable and reliable treatment processes [2]. Curcumin (CUR), which has a hydrophobic polyphenol structure, is an Indian herb and the main compound of turmeric; it has antioxidant, disinfectant, antimalarial, anti-inflammatory, and anti-cancer effects [3, 4].

Kuttan *et al*. first reported the anti-cancer properties of CUR via a clinical report. The antioxidant activity of CUR was evaluated by Weber *et al*. [5], and CUR treatment was found to overcome the stromal protection of chronic lymphocytic leukemia (CLL) B-cells in vitro. In the last few decades, the effects of CUR on cancer and complication of treatments were evaluated in several ways [5, 6, 7]. CUR can prevent cancer by elevating biomarkers, such as CD133, CD44, CD166, and ALDH1, which affect the morphology of cancer cells [8, 9]. Yallapu *et al*. used encapsulated CUR in poly(lactic-co-glycolide) (PLGA) nanoparticles to treat ovarian and breast cancer; their results demonstrated elevated anti-cancer effects of CUR in nanoencapsulated formulation [10]. In the present study, we aimed to increase the apoptosis effect of CUR on CML cell lines using curcumin-loaded chitosan–stearic acid nanogel (CUR–CSA), which is a component without side effects.

## Materials and Methods

### 1. Chemicals

CUR ((1E,6E)-1,7-bis(4-hydroxy-3-methoxyphenyl)-1,6-heptadiene-3,5-dione) was purchased from Riedel de Haen (Germany). Chitosan, 1- ethyl- 3 (3-dimethylaminopropyl) carbodiimide (EDC), N-hydroxysuccinimide (NHS), MTT (3-(4,5-dimethyl thiazolyl)-2, 5-diphenyl-tetrazolium bromide; Sigma Co., St. Louis, MO, USA), 1% (v/v) acetic acid (Merck, Darmstadt, Germany), and the K562 CML cell line was purchased from the National Cell Bank of Iran (NCBI). Fetal bovine serum (FBS) was purchased from Gibco (Rockville, MD, USA), and penicillin–streptomycin solution was obtained from Caisson Laboratories, Inc. (USA). Trypan blue stain was procured from Bio-Idea (Iran), and the equipment for flow cytometry was from IQ Products BV (Groningen, The Netherlands).

### 2. Preparation of the Nanodrug CUR–CSA

The nanodrug CUR–SCS was prepared according to the protocol of Atabi *et al*. [11] with slight modifications. In brief, 0.5 g of chitosan was dissolved in 100 ml of 1% (v/v) acetic acid, homogenized to achieve 5% chitosan solution (5 mg/ml), and sonicated in 60 kHz. Stearic acid was dissolved in methanol, and EDC and NHS were added to the chitosan solution. Ethanol was added to the solution and mixed in a dark room. Sodium hydroxide was added to the gel after 1 day and centrifuged at 5,000 rpm for 10 min. The precipitate was washed with distilled water and ethanol twice. The pellet was dissolved in 1% (v/v) acetic acid, sonicated in 60 kHz, and filtered by 0.22-micron filter. The obtained nanogel solution was stored in 4 °C for further use [11]. For loading CUR in nanogel, required amounts of CUR and nanogel were incubated in 15°C after sonication in 50 kHz.

### 3. Evaluation of Physicochemical Properties

Dynamic light scattering (DLS), transmission electron microscopy (TEM), and Fourier transform infrared (FTIR) spectroscopy were conducted to evaluate the properties of the chitosan nanogel and nanodrug.

#### 3.1. DLS

The mean particle size and particle size distributions of the nanogel and nanodrug were determined by a Zeta plus DLS Zeta Sizer Nano-ZS-90 (Malvern Instruments). The mean particle size was measured for 234 and 239 replicates for the nanogel and nanodrug, respectively. The polydispersity index (PdI) was also calculated. To disperse the nanoparticles, samples were sonicated in a water bath. The Zeta Plus instrument was used to measure the electrophoretic mobility of the SCS nanogel and nanodrug.

#### 3.2. TEM

TEM creates images with a higher resolution than a light microscope by transmitting electrons. With this technique, the smallest details of a particle can be seen. TEM images were obtained using a CM120 electron microscope (Philips, USA) equipped with a Tietz 2K × 2K CCD camera and a fiber optically coupled Gatan Orius 832 camera.

#### 3.3. FTIR

FTIR was used for detecting the interaction between different compounds of nanoparticles. Although the infrared spectrum specifies the molecular structure of a compound, some organic groups show a certain frequency. The frequency or wavelength of absorption depends on relative atomic mass, binding force constant, and geometry of atoms. For this purpose, CUR and nanodrug were mixed with pure KBr separately and then compressed to form tablets. The spectra of the samples were obtained using a Bruker VERTEX 70 (Germany) spectrophotometer.

### 4. Cell Culture

The K562 CML cell line was purchased from the NCBI. The cells were cultured, passaged in a 25 ml flask with RPMI 1640 medium, 10% FBS, and 1% penicillin–streptomycin solution, and placed in an incubator (VS-9160C; Bionex, Seoul, South Korea) in 37°C and 5% CO2 for 24 h before treating CUR and nanodrug.

### 5. Evaluation of Cell Viability

Trypan blue staining, MTT assay, and flow cytometry analysis were conducted to determine cell viability.

#### 5.1. Trypan Blue Staining

The level of cell viability by trypan blue staining was measured as described by Banerjee *et al*. [12]. In this method, blue and colorless cells represented dead and viable cells, respectively. The examination was performed in duplicate.

#### 5.2. MTT Assay

MTT assay was performed according to the protocol of Entezari *et al*. [13]. Living cells can convert yellow MTT to purple by the mitochondrial succinate dehydrogenase enzyme. The number of living cells after treating 1.5 × 104 cells per well with the drug was determined by MTT assay under an ELISA reader (ELx800, BioTek, Winooski, VT, USA) at a wavelength of 560 nm. The mortality and viability rates of cells were calculated with the following equation:

$$Cytotoxicty\%=1-\frac{Mean\;absorbance\;of\;toxicant}{Mean\;absorbance\;of\;negative\;control}*100$$

Viability%=100−Cytotoxicity%

#### 5.3. Flow Cytometry

Flow cytometry was performed according to Entezari *et al*. [13]. An Annexin V-FITC kit was used to evaluate primary and secondary apoptosis.

### 6. Statistical Analysis

Statistical analysis was performed using SPSS software version 16 (SPSS, Inc., Chicago, IL, USA). Data are expressed as the mean ± SD. Statistical comparison was calculated using Tukey’s test. Statistically significant differences were indicated by P< 0.05.

## Results

### 1. DLS

**Figure-1:**
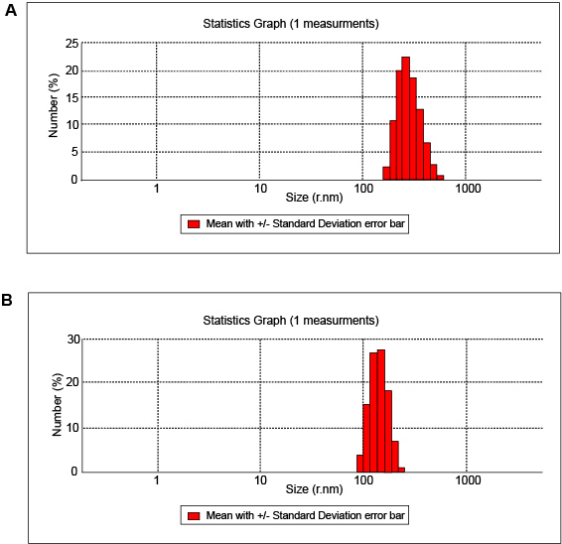
DLS Results for (A) Nanogel and (B) Nanodrug

**Figure-2:**
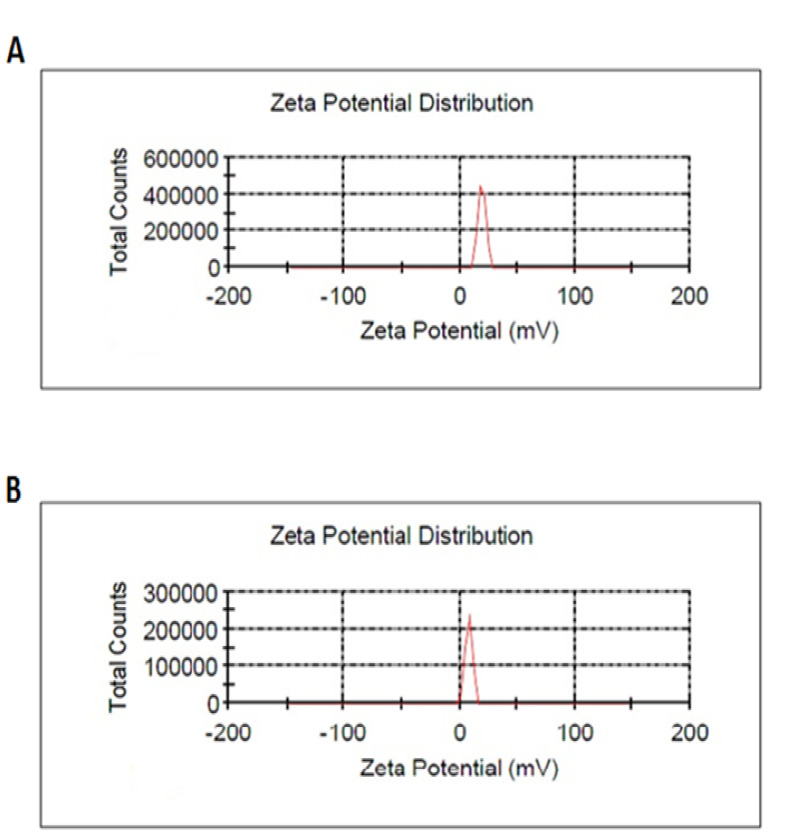
Zeta Potential Distribution Results for (A) Nanogel and (B) Nanodrug

Table-1 shows the results of the nanogel and nanodrug characterized by DLS. Figures-1 and 2 show the particle size of the nanogel and nanodrug characterized by DLS analysis, respectively. The average diameters of the nanogel and nanodrug were 287.306 and 167.1 nm, respectively. Zeta potentials of the nanogel and nanodrug, which were determined at 25 °C, are given in Table-1 and Figure-2, respectively. The PdIs obtained for the nanogel and nanodrug were 0.279 and 0.147, respectively.

**Table-1:** DLS of Chitosan–Stearate Acid Nanogel and Nanodrug

**Sample**	**Nanogel** **(CSA)**	**Nanodrug** **(CUR-CSA)**
**Mean particle size by DLS**	278.306	167.1
**DLS record number**	234	239
**Zeta potential (mV)**	19.4 ± 3.21	7.93 ± 2.97
**PDI**	0.279	0.147

### 2. TEM

**Figure-3:**
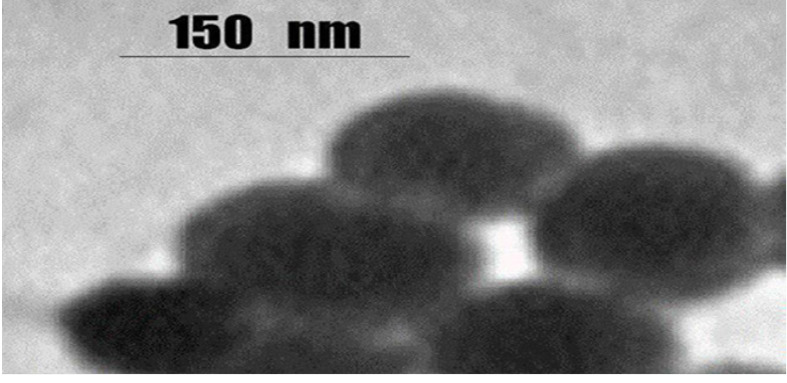
TEM Scan of Nanodrug Particles. The Size of The Nanodrug was Between 150 and 200 nm. TEM Images were Obtained Using CM120 Electron Microscope (equipped with a Tietz 2K × 2K CCD camera and a fiber optically coupled Gatan Orius 832 camera).

Estimates of the shape and size of the nanodrug via TEM are shown in Figure-3. The nanodrug presented a smooth surface on the TEM image. The size of the nanodrug was between 150 and 200 nm.

### 3. FTIR

**Figure-4:**
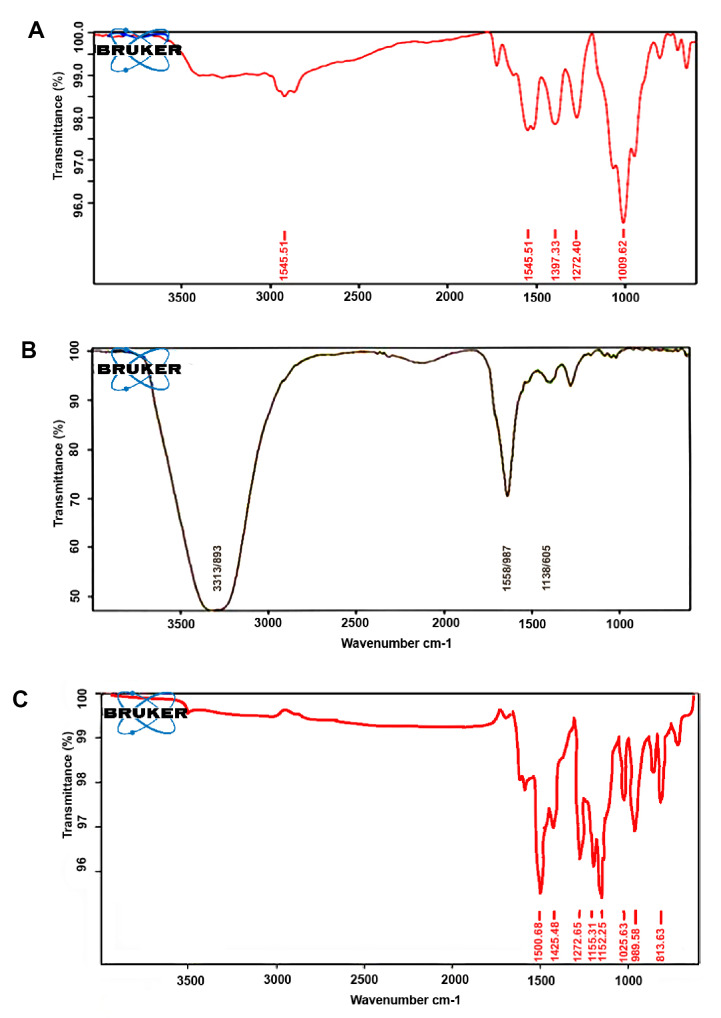
FTIR Results of The **(A)** Nanodrug, **(B)** Nanogel, and **(C)** CUR (More Details in the Text).

The results of the structural assessment of nanodrug, nanogel, and CUR evaluated via FTIR spectrometry are shown in Figure-4. The peaks of the nanodrug (Figure-4A) at 1009, 1272, 1548, 1700, and 2921 cm^-1^ were related to C–O stretching, C–C stretching, C=C stretching, C=O carbonyl stretching, and O–H stretching, respectively. In Figure-4B, the peaks of the nanogel were observed at 1438 and 1658 cm^−1^, and a strong broad peak was found at 3313 cm^−1^. The absorption bands at 1025, 1272-1152, 1500, and 1600 cm^−1^were observed in CUR (Figure-4C).

### 4. Trypan Blue Staining

**Figure-5:**
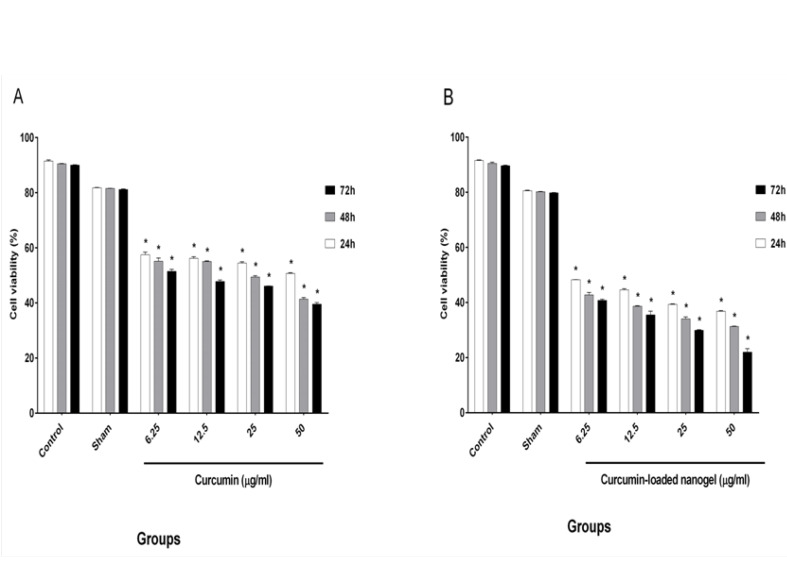
Trypan blue dye exclusion test for viability of K562 cells. Treatments (24, 48, and 72 h): control, 6.25, 12.5, 25, and 50 μg/ml curcumin **(A)** and curcumin-loaded nanogel **(B)**. These figures demonstrate that the curcumin-loaded nanogel reduced viability of K562 cells more than curcumin in a time- and dose-dependent manner. Significant difference from the control was indicated by *P < 0.05.

Cell viability after treatment with four selective doses of CUR and nanodrug was evaluated by trypan blue staining, and the results are presented in Figure-5.

The cells were counted, and the half maximal inhibitory concentration (IC_50_) was calculated for varying concentrations of the nanodrug (24 h: 6.25 ± 0.07 μg/ml, 48 h: 6.25 ± 0.84 μg/ml, and 72 h: 6.25 ± 0.42 μg/ml) and CUR (24 h: 50 ± 0.14, 48 h: 25 ± 0.49, and 72 h: 6.25 ± 0.70).

### 5. MTT Assay

**Figure-6:**
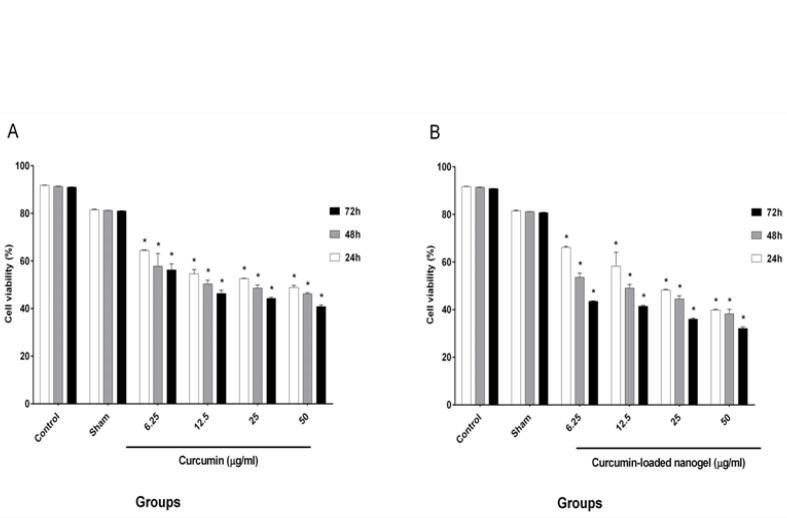
The MTT assay depicte the inhibitory concentrations for the K562 cell line in vitro.Varying doses of curcumin **(A)** and curcumin-loaded nanogel **(B)** were administered to K562 cells for 24, 48, and 72 h. Cell viability was analyzed by MTT assay. Treatment with curcumin and curcumin-loaded nanogel significantly decreased viability in K562 cells (*P < 0.05) compared with the control (untreated K568). Data are expressed as the mean ± SD.

The results of cell viability, which was evaluated by MTT assay, after treatment with four selective doses of CUR and nanodrug are shown in Figure-6.

IC_50_ was obtained for CUR (24 h: 50 ± 0.84 μg/ml, 48 h: 25 ± 1.34 μg/ml, and 72h: 12.5 ± 2.54 μg/ml). The results for the nanodrug were 25 ± 0.28, 12.5 ± 1.48, and 6.25 ± 0.21 μg/ml for 24, 48, and 72 h, respectively.

### 6. Flow Cytometry

**Figure-7:**
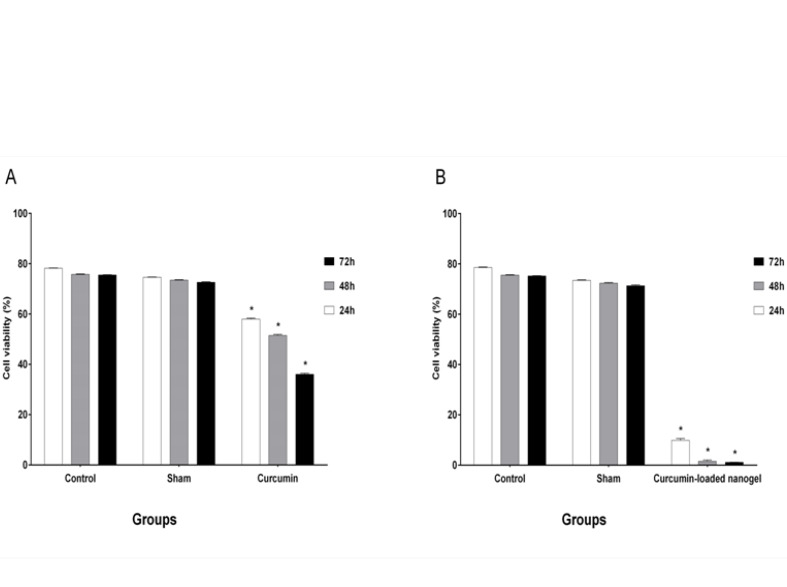
Viability of K562 cells after treatment with 50 μg/ml curcumin and curcumin-loaded nanogel in different times by flow cytometry. Treatment with curcumin **(A)** and curcumin-loaded nanogel **(B)** significantly decreased viability in K562 cells (*P < 0.05) compared with the control (untreated K568). Data are expressed as the mean ± SD.

**Figure-8:**
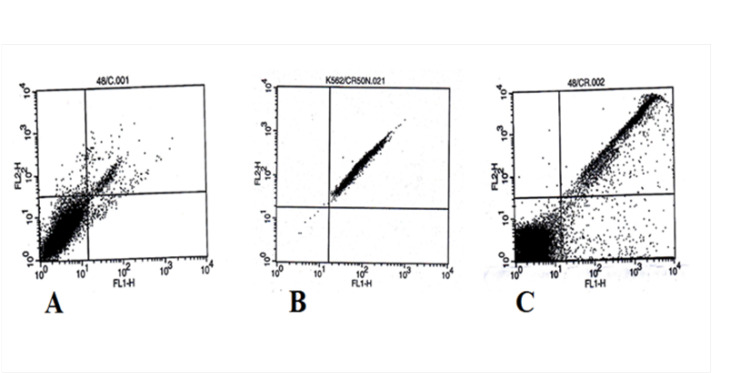
Apoptotic effects of curcumin and curcumin-loaded nanogel (50 μg/ml) on the K562 cell line at 48 h by flow cytometry. **A**: Untreated cells; **B**: Curcumin-loaded nanogel; **C**: Curcumin

The apoptosis rates in the K562 cell line after treatment with 50 μg/ml CUR and nanodrug CUR–SCS were evaluated by flow cytometry, and the results are shown in Figures-7 and 8. The apoptosis rates for CUR at 24, 48, and 72 h were 35%, 48%, and 60%, respectively. The apoptosis rates for the nanodrug were 93%, 96%, and 99% at 24, 48, and 72 h, respectively.

## Discussion

CML is one of the basic neoplastic deviations connected to a genetic abnormality known as Philadelphia chromosome, and it occurs in the presence of the BCR-ABL1 oncogene. Philadelphia chromosome is caused by a translocation between chromosomes 9 and 22 and activated by tyrosine kinase [14, 15]. Different treatments have produced many side effects and toxicities in patients. Conventional methods for treating various types of cancers can produce side effects and damage healthy and cancerous cells. Thus, appropriate treatment with minimal side effects is necessary. CUR is an active polyphenol derived from Curcuma longa L. roots, and their multiple features are approved [16, 17]. CUR is a safe herbal drug that has shown several unique features during cancer treatment. These features include lack of toxicity, low price, availability, and novel mechanisms for cancer treatment [18]. The prevention effects and treatment of cancers with CUR on humans have been confirmed. Low solubility in water and susceptibility to physiologic pH are the main problems of CUR [16, 19]. In this study, we prepared the nanodrug CUR–CSA by inserting the drug into the nanogel via sonication. Stearate was used as a hydrophobic media for the nanogel, and CUR was then attracted into the nanogel. In the IR spectrum of the nanogel, a broad and sharp peak was observed at 3313 cm-1 in the main structure of chitosan, and this peak was due to the frequency vibrations of N-H, C-H, and O-H. Thus, the presence of stearate in the nanogel structure was confirmed.

According to Figure-4, the peak at 3313 cm^−1^ in the nanogel, which was attributed to the O-H group, shifted to 2921 cm^−1^ in the nanodrug due to the presence of CUR in the nanogel polymer. This change may also be the result of the intramolecular hydrogen bond between the O-H groups in CUR and chitosan. The results of FTIR analysis in this study were consistent with the findings of Abd El-Rahman *et al*., who reported that the drug does not interact with the nanogel polymer structure, and CUR can remain in the nanogel without any structural changes. The absorption band in 1009–1272 cm^−1^ of the nanodrug CUR–CSA represented flexural vibrations in the methyl group, which were consistent with the findings of Bisht *et al*.

Entezari *et al*. used myristoylated chitosan nanogel as a carrier for treating breast cancer. They reported that the myristoylated chitosan nanogel can be used as a carrier with a high loading capacity and minimal side effects in cancer treatment [20].

Karmakar *et al*. experimented on glioblastoma cells and showed that cell viability increases with a decline in CUR concentration. They treated cells with different CUR concentrations (25 and 50 μM) for 24 h. The viability rates of cells in 25 and 50 μM CUR were 75% and 50%, respectively [21]. In our study, the amounts of viable cells decreased under high CUR concentrations, which indicated that the effects of CUR were dependent on the concentration. However, 50% of the cells were viable under 50 μg/ml CUR for 24 h, and this rate reached 40% in 48 and 72 h. Our results were consistent with the findings of Karmakar *et al*. [21]

Bisht *et al*. [22] demonstrated the effect of CUR and polymeric nanoparticles loaded with CUR on stellate cells, and the effects of free CUR and nano CUR were almost the same. They treated cells with 20, 40, and 80 μM CUR and nano CUR, and the percentage of cell viability was determined by trypan blue assay. The rates of cell viability for 20, 40, and 80 μM CUR were 75%, 20%, and 0%, respectively. These values were similar to those obtained under the same concentrations of CUR-loaded nanogel (80%, 30%, and 0%) [22]. In the present study, the effects of CUR and nanodrug on the K562 cell line were evaluated. The toxicities of the nanodrug and CUR increased in a time- and concentration-dependent manner. Our resalts shows that cells were treated with 6.25, 12.5, 25, and 50 μg/ml CUR and nanodrug for 24 h. The cell viability in 50 μg/ml CUR and 6.25 μg/ml nanodrug was 50%. This result showed the improved efficacy of the CUR-loaded nanogel compared with free CUR. The effect of CUR and liposomal CUR on the B16F10 cell line was evaluated by Karewicz *et al*. [23] using 2.5, 5, 10, 20, and 30 μM CUR and liposomal CUR. Their results demonstrated that the toxicity of liposomal CUR up to 10 μM exceeds that of CUR alone, but an opposite trend was found in higher concentrations. In 10 and 20 μM CUR, the rates of the viable cells were 94% and 70%, respectively; under the same concentration of liposomal CUR, 82% and 77% of cells survived, respectively [23]. In the present study, the use of the chitosan–stearate nanogel solved the problem of CUR toxicity. According to Figure-6, 25 μg/ml nanodrug for 24 h induced apoptosis in half of the cells. The use of 12.5 μg/ml nanodrug for 48 h induced apoptosis in less than half of the cells, and 6.25 μg/ml nanodrug for 72 h induced apoptosis in more than half of the cells. In comparison with the results of Karewicz *et al*., unlike liposomal CUR, the CUR-loaded nanogel could decrease the rate of the surviving cells in high concentrations.

The effects of CUR and CUR-loaded micelles on glioma cells were determined by Zheng *et al*. They measured the apoptosis rate using an annexin kit and treated cells with 3.125, 6.25, and 12.5 mg of CUR and CUR-loaded micelles for 24 h. At 3.125 μg of CUR and CUR-loaded micelles, apoptosis was observed in 10.79% and 15.24% of the cells, respectively. Apoptosis occurred in 15.45% and 18.7% of the cells treated with 6.25 μg of CUR and CUR-loaded micelles, respectively. By contrast, the apoptosis rates for 12.5 μg of CUR and CUR-loaded micelles were 20.51% and 51.22%, respectively. These results demonstrated that the effect of CUR-loaded micelles in the induction of apoptosis in glioma cells was stronger than that of CUR [24]. In our work, the K562 cell line was treated with 50 μg/ml CUR and nanodrug for 24 h, and we observed 35% and 93% of apoptosis, respectively. The apoptosis rates were 48% and 96% at 48 h, respectively, whereas those for CUR and nanodrug were 60% and 99% at 72 h, respectively.

## Conclusion

We describe the encapsulation of CUR in CSA nanogel on the K562 cell line. Results showed that the toxicities of the CUR and CUR-loaded nanogel (CUR–CSA) were elevated in a time- and concentration-dependent manner. Furthermore, a significant difference between the induction of apoptosis in K562 cells by CUR and CUR-CSA was observed. CUR–CSA was more effective than free CUR and presented a solution against the problems of CUR. It could also be effective at low doses. This nanogel CSA could be considered a carrier in anticancer treatment.

## Conflict of Interest

The authors report no conflict of interests in this article.
